# Quality by Design in Downstream Process Development of Romiplostim

**DOI:** 10.52547/ibj.3790

**Published:** 2022-10-30

**Authors:** Saeedeh Pouri, Fatemeh Torkashvand, Hooman Aghamirza Moghim, Pezhman Fard-Esfahani, Majid Golkar, Behrouz Vaziri

**Affiliations:** 1 Biotechnology Research Center, Pasteur Institute of Iran, Tehran, Iran;; 2 Department of Biochemistry, Pasteur Institute of Iran, Tehran, Iran;; 3 Department of Parasitology, Pasteur Institute of Iran, Tehran, Iran

**Keywords:** Romiplostim, CQA, Design space, Quality by Design, risk assessment

## Abstract

**Background:**

Downstream processing of therapeutic recombinant proteins expressed as the IBs in *E. coli* is quite challenging. This study aimed to use the QbD approach for developing the multi-step downstream process of a structurally complex therapeutic Fc-Peptide fusion protein, romiplostim.

**Methods:**

For development of a successful downstream process, risk analysis and experimental designs were used to characterize the most CQAs and effects of process parameters on these quality attributes.

**Results:**

The solubilization of IBs was optimized by DoE on three parameters with a focus on solubility yield, which resulted in >75% increase of the target protein solubilization. The pH of sample was identified as CQA in AEX that might have an impact on achieving >85% host cell proteins removal and >90% host cell DNA reduction. In the refolding step, process parameters were screened. Cystine/cysteine ratio, pH, and incubation time identified as CPPs were further optimized using Box-Behnken analysis, which >85% of the target protein was refolded. The design space for further purification step by HIC was mapped with a focus on HMW impurities. After polishing by gel filtration, the final product's biological activity showed no statistically significant differences among the groups received romiplostim and Nplate^®^, as the reference product.

**Conclusion:**

This research presents a precise and exhaustive model for mapping the design space in order to describe and anticipate the link between the yield and quality of romiplostim and its downstream process parameters.

## INTRODUCTION

To design and develop well-characterized pharmaceutical processes, which consistently deliver products with predefined qualities, the QbD approach is strongly suggested^[^^1^^]^. The QbD utilizes risk analysis, statistical methods, and experimental design to gain an in-depth understanding of the effects of process parameters on each other and product quality^[^^2^^,^^3^^]^. QbD approaches are used in the product development processes of different proteins expressed in *E. coli* and have successfully been utilized to optimize the upstream process^[^^4^^]^ and refolding^[^^5^^,^^6^^]^. QbD is also used to create a model that identifies CPPs for HIC^[^^7^^]^.

Using QbD approach, the current study optimized the downstream processing of romiplostim, a therapeutic Fc-fusion protein with a complex structure^[^^8^^]^. A molecule contains two identical single-chain subunits, each consisting of human immunoglobulin IgG1 Fc domain fused to a peptide with two thrombopoietin receptor binding domains^[^^8^^]^. CQAs were identified by risk ranking and filtering for mapping the process design space. The prior/platform knowledge, laboratory data along with clinical and nonclinical data on romiplostim or other similar molecules were considered information sources. Process parameters in each step were determined either by prior knowledge or literature searching. The impact of these parameters on CQAs was examined by the DoE to identify the CPPs. The results were analyzed by statistical methods to determine the influence of each CPP, alone or in interaction with others. Using the related CQAs, The design space for each step was determined, and the proper results were achieved based on yield and quality. This approach helped to define and predict the impact of different process parameters on yield and quality of romiplostim and could be used for other therapeutic proteins expressed in *E. coli*.

## MATERIALS AND METHODS

**CQAs determination**

Risk ranking and filtering was used to evaluate CQAs of romiplostim regarding its safety, pharmacokinetics and pharmacodynamics, immunogenicity, and efficacy^[^^9^^]^. The effect and uncertainty of each factor were ranked as 2-20 and 1-7, respectively. A RPN was calculated by multiplying the impact score by uncertainty score. Filtering of the risks was performed using cut-off values for scores^[^^9^^]^ to identify high-risk (CQAs) and low-risk (non-CQAs) attributes (Supplementary Table 1). The uncertainty around the impact ranking was based on the relevance of information used to assign the impact ranking (Supplementary Table 2). The highest RPN in each category determined the overall risk score for the quality attribute.

Expression of recombinant romiplostim in *E. coli*

The expression plasmid was transfected into BL21(DE3) *E. coli* strain^[^^10^^,^^11^^]^. A single colony was inoculated into shake flasks containing 10 mL of Luria-Bertani liquid medium and 50 µg/mL of kanamycin and then cultured at 37 °C with 140 rpm rotation speed. This 10-ml culture inoculated 500 mL of Luria-Bertani medium in a baffled Erlenmeyer flask. At OD_600_ of 0.8, Isopropyl-β-D-Thiogalactopyranoside was added to a final concentration of 0.3 mM. Cells were cultivated for 6 h and then harvested by centrifugation at 8000 ×g at 4°C for 10 min.

SDS-PAGE and Western blotting

Protein samples were loaded onto the wells of 12% SDS-PAGE gel along with molecular weight markers (Sigma-Aldrich St. Louis, USA), and electrophoresis was run at 150 V for 1.5 h. The gels were either stained with Coomassie blue G250 (Sigma-Aldrich St. Louis) or transferred to a nitrocellulose membrane (Bio-Rad, USA). The membrane was blocked in 2.5% blocking solution (Tris-buffered saline containing 0.1% Tween-20 and 2.5% skim milk) at 4 °C overnight. Then the membrane was incubated with goat anti-human Immunoglobulin G Fc fragment specific, Horse radish peroxidase conjugate (Chemicon^®^) with 1:2000 dilution in 2.5% blocking solution at room temperature with mild agitation. After washing with Tris-buffered saline and 1% Tween-20 for three times, enhanced chemiluminescence was used to detect protein by film^[^^12^^]^.

Optimization of solubilization

The bacterial pellet was resuspended in the homogenization buffer (20 mM of Tris-HCl, 5 mM of EDTA, and 1% v/v Triton-X_100_, pH 8) at room temperature for 45 min. Cell disruption was performed by high-pressure homogenization at 800 bars for three passages; the homogenate was centrifuged at 8000 ×g at 4 °C for 25 min. The supernatant was discarded, and the remaining IBs with cell debris were washed three times with washing buffer (20 mM of Tris-HCl and 5 mM of EDTA, pH 8). The 50-mg pellets were solubilized under extreme condition (7 M of urea, 2 M of thiourea, 4% 3-cholamidopropyl dimethylammonio 1-propanesulfonate, and 50 mM of DTT). Moreover, the total amount of recombinant protein was determined by densitometry analysis of the related band on SDS-PAGE of solubilized pellet using Quantity One software (Bio-Rad). DTT concentration, incubation time, and urea concentration were selected as parameters for DoE, based on literature and previous experiences^[^^13^^]^. Fifteen sets of experiments have resulted from Box-Behnken^[^^14^^]^ experimental design (Supplementary Table 3). The amount of total protein obtained at each experiment was determined by Bradford assay. Densitometry analysis was used to assess the amount of romiplostim recovered during the solubilization process (solubilization yield) by determining the percentage of romiplostim band in each lane of non-reduced SDS-PAGE. 

**Anion** **exchange chromatography**

Flowthrough mode AEX was used to remove the process-related impurities such as HCD and HCP. The column was equilibrated with five CVs of binding buffer (20 mM of Tris-HCl, 20 mM of NaCl, and 1 mM of DTT). Then 6 mg of proteins were loaded on the column under the desirable conditions selected in the previous step. The flowthrough peak collection was started with a linear 1 ml/min flow rate of binding/equilibration buffer. The column was stripped with 3-5 CVs of 1.5-M NaCl and 2-4 CVs of 0.5-N NaOH, respectively. Host-cell DNA and host-cell protein were measured by HCD and HCP ELISA kit assays (Pico Green®, USA). The effect of pH (6.8, 7.4, and 8) on this process was studied. Protein dynamic binding capacities on ion exchange resins were typically expected to decrease with increasing conductivity and reducing protein charge^[^^15^^]^. Therefore, the ionic conductivity was set at the lowest value of about 2 mS/cm.

Optimization of refolding

Plackett-Burman's design^[^^16^^]^ was performed at 12 sets of the experiment to screen seven selected refolding process parameters (Supplementary Table 4). The process parameters and their limits were selected based on the previous studies in the literature^[^^17^^,^^18^^]^. The output was refolding yield that was determined by densitometry analysis of non-reduced SDS-PAGE gradient gel. After selecting the CPPs, Box–Behnken experimental design was employed to optimize these parameters based on 15 sets of experiments. Utilizing analytical reverse-phase HPLC, the effects of process parameters on refolding yield and process contaminants such as oxidized forms were evaluated by an analytical C4 column (250 mm × 4.6 mm; 5 m; YMC-Pack, Japan) with a linear gradient of 0.1% *Trifluoroacetic acid* in acetonitrile (from 10% to 100%) at a flow rate of 1 mL/min for 60 minutes. The refolding yield was calculated as the AUC of romiplostim main peak to the sum of AUC of all peaks present in the chromatogram. The reference product, Nplate®, was analyzed by the same method to determine the main peak. The oxidized forms were generated by 2-h incubation with hydrogen peroxide (0.1% v/v) to identify the peak of the oxidized impurities. AUC in each chromatogram of the experiment was calculated and subtracted from the percentage of the main peak and oxidation percentages as impurities.

Optimization of HIC

Based on the literature and previous studies, three influencing parameters (pH, ammonium sulphate concentration, and urea concentration) were selected^[^^19^^,^^20^^]^. Box-Behnken design was employed to optimize these parameters on 15 sets of the experiments (Supplementary Table 5). On a Biologic LP chromatographic system, all chromatographic studies were conducted (Bio-Rad). Five milligrams of the refolded sample were put to a phenyl Sepharose high-performance column (GE Healthcare, Switzerland) that had been equilibrated with five concentration units (CVs) of binding buffer (50 mM of phosphate-buffered saline, 2 mM of EDTA, and 2 M of urea). The procedure was applied using a stepwise gradient at 1 mL/min flow rate. This exercise enables the identification of factors influencing important outcomes from the HIC (e.g., protein purity, protein recovery, and HMW aggregate). The purity of protein obtained from each experiment was determined by densitometry analysis of the samples on non-reduced SDS-PAGE. Aggregate content in the samples was determined using SEC performed with Tosoh TSK 3000 SW XL 7.8 × 300 mm column (Tosoh Bioscience LLC, Part No. 08541, King of Prussia, PA, USA). The mobile phase consisted of 100 mM of ammonium hydrogen carbonate, pH 7. The analysis was performed in isocratic mode with 1 ml/min-flow rate at 40 min. In order to final polishing and buffer exchange, preparative SEC was performed with Superdex 75 (Hiload^TM^ 16/600, Cytiva, Sweden) in isocratic mode with 0.5 ml/min-flow rate for 140 min. The mobile phase consisted of 150 mM of NaCl, 50 mM of CH_3_COONa.3H_2_O, and 0.5 M of urea, pH 5.5. 

Potency assay

Female BALB/c mice (n = 95, 8-9 weeks old) were housed in plastic cages with free access to tap water and standard rodent pellets under a constant 12:12 h light-dark cycle. Two main groups received different subcutaneous doses of Nplate^®^ (Amgen, Thousand Oaks, CA) or romiplostim. Each main group was divided into three subgroups, which received different subcutaneous doses (1, 10, and 100 μg/kg). Each subgroup included three sets of five mice. A control group of five mice was sampled to determine the baseline platelet count. On days 1, 3, and 5, the whole blood was collected through preorbital sinus sampling, and blood cells were counted by a hematology analyzer (Sysmex KX-21). 

**Data analysis**

 Data analysis Design-Expert® software (version 13.0.0, Stat-Ease, Inc., Minneapolis, USA) was used to analyze obtained data. The best-fitting mathematical model was selected based on the comparisons of several statistical parameters, including the determination coefficient (R2), the adjusted determination coefficient (adj-R2), and the F-value/p -value provided by analysis of variance (ANOVA).

## RESULTS

Determination of CQAs

For each quality attribute, the highest RPN of potency, pharmacokinetics and pharmacodynamics, immunogenicity, and safety was selected as the definite RPN (Table 1). The quality attributes with RPN higher than a pre-defined threshold (12) was considered a potentially CQA. Risk study identified HMW aggregation, host cell proteins, host cell DNAs, oxidized forms, deamidation forms, and the right disulfide connections as crucial quality aspects of romiplostim (CQAs). 

**Expression of romiplostim**

The recombinant plasmid was transformed into *E. coli* BL21 strain. Expression of the fusion protein was induced by IPTG, followed by cultivation for 6 h. SDS-PAGE analysis showed the presence of a protein with a molecular weight of about 30 kDa in induced bacteria (Supplementary Fig. 1). Western blot analysis was performed to identify the target protein. Anti-human Fc monoclonal antibody recognized romiplostim as well as standard (Supplementary Fig. 2).

Optimization of solubilization

**Fig. 1:**
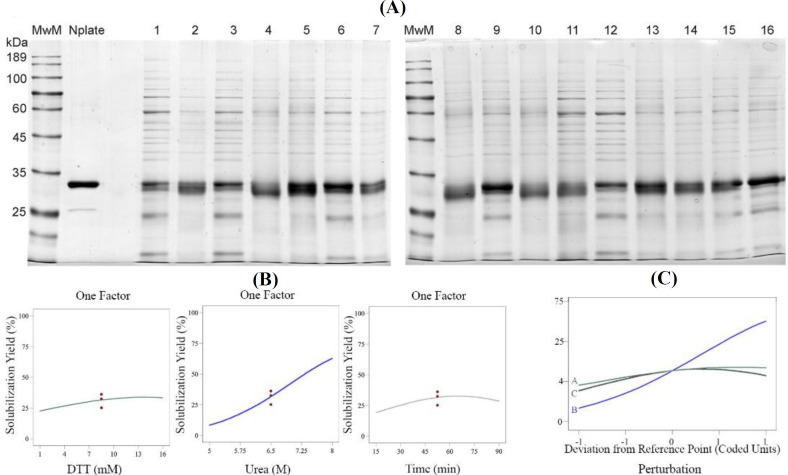
Optimizing of IB solubilization by Box-Behnken. (A) Non-reduced 12% SDS-PAGE of the designed 15 experiments. The gel was stained with Coomassie brilliant blue (G-250). The reduced form of Nplate® was used as a reference product. Lane 16 is the reduced form of sample 13 for the assessment of the aggregation profile; (B) the effect of process parameters (DTT, Urea, and incubation time) on the solubilization yield. DTT, urea and incubation time were known as CPPs and had a positive effect on the solubilization yield of romiplostim; (C) perturbation plot of DoEs for verification

Box-Behnken experimental results shown in Table 2, and obtained from SDS-PAGE (Fig. 1A), were further analyzed by Design-Expert software (Supplementary Table 6). The significance of the proposed model for the response of solubilization yield was indicated by F-value of 24.91 and a low probability *p* value ≤ 0.05. The proposed model for this response was expressed as a polynomial equation, Eq. (**1**), in terms of three variables, in which A, B, and C are coded values of DTT, urea, and incubation time, respectively. 

Eq. (1): 

Solubilization Yield = 1.490 + 0.085A+ 0.438B + 0.0862C + 0.0535AB + 0.006AC + 0.027BC-0.0611A2-0.1406B2-0.1296C2

The coefficient of determination (R^2^: 0.97) determines the goodness of fit for the model. Through the given model, the influence of each process parameter on the solubilization yield was evaluated individually (Fig. 1B). The effect of DTT, urea, and incubation time on solubilization yield was significant (*p* value ≤ 0.05), and the perturbation plot helped compare the effects of all the factors at a particular point in the design space (Fig. 1C). According to the prediction model, Eq. (**1**), the highest solubilization yield of the predicted value (78.2%) was very close to the actual value (79.2%). For validation of the optimum point, duplicate experiments were conducted using the optimized parameters obtained.

**Table 1:** Box-Behnken matrix

**Run**	**DTT (mM)**	**Urea (M)**	**Time (min)**	**Solubilization yield (%)**
1	8.5	5.0	90.0	6.4
2	8.5	6.5	52.5	32.4
3	16.0	5.0	52.5	9.0
4	1.0	6.5	15.0	14.9
5	8.5	8.0	15.0	38.0
6	16.0	6.5	90.0	27.4
7	16.0	6.5	15.0	16.8
8	1.0	8.0	52.5	32.8
9	16.0	8.0	52.5	79.2
10	1.0	6.5	90.0	23.0
11	1.0	5.0	52.5	6.1
12	8.5	5.0	15.0	5.2
13	8.5	8.0	90.00	60.0
14	8.5	6.5	52.5	25.2
15	8.5	6.5	52.5	36.2

The output of each run is expressed as solubilization yield. Fifteen runs were designed at various combinations of values of solubilization process parameters.

**Table 2:** Critical and non-CQAs of the Fc-Fusion protein identified through the risk assessment

**Quality Attribute**	**Biological activity/ efficacy (I × U)**	**PK/PD** **(I × U)**	**Immunogenicity** **(I × U)**	**Safety** **(I × U)**	**RPN**
Disulfide linkages	20 × 3	20 × 3	20 × 3	20 × 3	60
Aggregated forms	16 × 3	16 × 3	16 × 3	20 × 3	60
Deamidated forms	16 × 3	16 × 3	16 × 3	2 × 7	48
Oxidized forms	16 × 3	16 × 3	16 × 3	2 × 7	48
HCP	2 × 3	2 × 3	12 × 3	12 × 3	36
HCD	2 × 3	2 × 3	2 × 3	12 × 3	36
Endotoxins (LPS)	2 × 1	2 × 1	2 × 1	12 × 1	12

The highest I × U among different categories (range between 2 and 140) defines the RPN. The cut-off score of 12 was considered for filtering the critical from non-CQAs. PK, pharmacokinetics; PD, pharmacodynamics

Optimization of AEX 

The pI of romiplostim is 8.2 (Supplementary Fig. 3); therefore, AEX with Q Sepharose FF in a flowthrough mode was used to purify romiplostim. Consequently, the pH of loading sample was identified as CPP in this step, which might have an impact on achieving >85% HCP removal and > 90% HCD reduction. At basic conditions, the positive charge of protein decreased, and the recovery value was low. The purity of target protein at different pH ranges was the same (Supplementary Fig. 4). Among the three pHs examined, pH 6.8 had the highest recovery yield with an acceptable HCD and HCP range (Table 3).

Optimization of refolding 

The effects of cystine/cysteine ratio, L-arginine, pH, EDTA, urea, incubation time, and protein concentration on refolding yield were studied by a Plackett-Burman design (Table 4). The significance of the proposed model for refolding yield was indicated by the F-value of 5.52 and a low probability value *p* ≤ 0.05. The model fit quality was evaluated by analysis of variance (ANOVA) and coefficient of determination. The proposed model for this response was expressed as a polynomial equation Eq. (**2**) in terms of five variables, in which A, B, C, F, and G are coded values of pH, incubation time, cystine, urea, and protein concentration, respectively.

Eq. (2):

Refolding yield = 57.31 + 22.05A + 0.6167B - 15.68C + 7.59F - 5.23G

The coefficient of determination (R^2^: 0.82) revealed the goodness of fit for the model. Cystine concentration and pH (*p* value ≤ 0.05) were identified as CPPs, having the potential effect on the refolding yield (Supplementary Table 7). Based on contribution percentage and effect, urea concentration and incubation time showed positive, and protein concentration exhibited a negative effect on the refolding yield (Supplementary Fig. 5). Cystine/cysteine ratio, pH, and incubation time were further optimized using Box-Behnken analysis. Regarding the positive effect of incubation time, a broader time range was selected for this experiment (Supplementary Fig. 6), while the concentration of urea and protein was kept at the highest and lowest limit, respectively. ANOVA was carried out to analyze the results of Box–Behnken (Table 5). The significance of the proposed model was indicated by the F-value of 6.6 and a *p* value ≤ 0.05 (Supplementary Table 8). The proposed model for the refolding yield was expressed as a polynomial equation Eq. (3) in terms of three variables, in which A, B, and C are coded values of pH, cystine/cysteine ratio, and incubation time, respectively.

**Table 3:** Experimental details for HCP and HCD assay at 2 mS/cm conductivity

**Sample**	**pH**	**Protein** **recovery (mg)**	**DNA** ^*^ ** (ng/dose)**	**HCP** **(ng/mg)**
Soluble	8.0	-	59.7	3139.7
1	6.8	3.1	5.4	337.8
2	7.4	2.6	5.6	330.5
3	8.0	0.6	23.0	716.4

^*^Each dose contains 250 µg of romiplostim.

Eq. (3):

Refolding yield = 51.13 - 1.85A + 13.94B + 10.81C - 5.62AB-9.52AC + 3.3BC + 0.3583A^2 ^+ 5.08B^2 ^+ 1.38C^2^

The coefficient of determination (R^2^: 0.92) revealed the goodness of fit for the model. The significance of the proposed model for oxidized forms was indicated by the F-value of 15.33 and a *p* value ≤ 0.05 (Supplementary Table 9). The proposed model for the oxidized forms was expressed as a polynomial equation Eq. (4) in terms of three variables, in which A, B, and C are coded values of pH, cystine/cysteine ratio, and incubation time, respectively.

Eq. (4): 

Oxidized form = 18.27 + 0.8A-2.99B - 3.31C + 4.2AB + 1.85AC - 0.125BC - 1.47A^2 ^- 0.0958B^2 ^- 3.25 C^2^

**Fig. 2:**
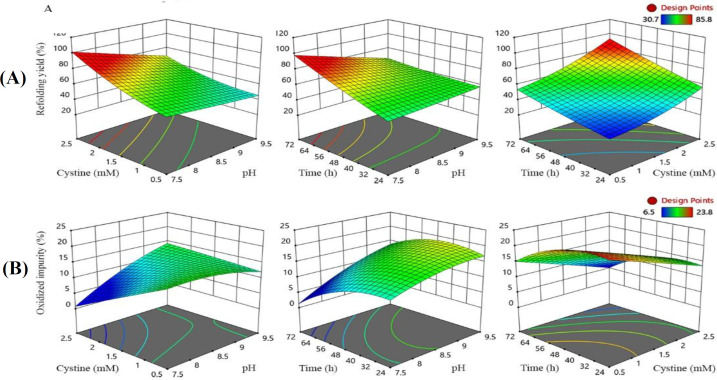
Quadratic RMS plots of CPPs and CQAs responses. Three dimensional response surface methodology RSM graph (A) refolding yield and (B) oxidized impurity. The effect of CPPs (pH, Cystine concentration, and time) at responses is shown as color-coding indicating high (red) to low (blue). Refolding yield was strongly dependent on the cystine/cysteine ratio, with a higher ratio leading to over twofold increase in refolding yield. The refolding yield increased with the raising incubation time, and pH decreased significantly. The oxidized forms of romiplostim decreased significantly with increasing the cystine/cysteine ratio in the lower values of pH

**Fig. 3:**
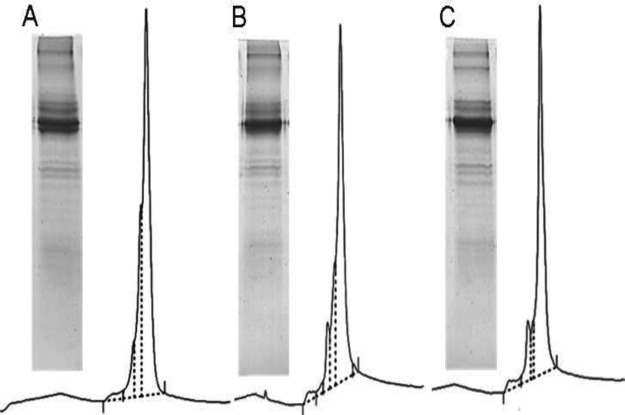
Comparison of SDS-PAGE analysis and RP-HPLC chromatograms of three out of the 15 experiments performed to optimize the refolding step. Experiment (A) no. 14. (B) no. 4, and (C) no. 5 with cystine/cysteine ratio of 0.3, 0.5, and 0.5, pH 8.5, and incubation time of 48, 24, and 72 h, respectively. Experiment no.5 showed the highest amount of correctly-refolded protein and the least impurities

The coefficient of determination (R^2^: 0.96) revealed the goodness of fit for the model. The optimum levels of process parameters were found at pH 7.5, cystine concentration of 2.4 mM, and incubation time of 59 h resulting in a maximum refolding yield of 88% and minimum oxidized forms of 6%. A three-dimensional surface graph was drawn to show the interactions between two parameters while keeping the third parameter at the optimum level for the combined outputs of refolding yield and oxidized impurities. The optimized range of process parameters led to refolding yield of ≥ 85% (Fig. 2A) and oxidized impurities of ≤10% (Fig. 2B). For validation of the optimum point, duplicate experiments were conducted using the optimized parameters obtained. The interaction of CPPs was crucial in correct folding and preventing the formation of misfolded and aggregated entities (Supplementary Fig. 7). SDS-PAGE and RP-HPLC results of three selected experiments, have been compared in Figure 3. 

Optimization of HIC 

The effects of pH, ammonium sulphate, and urea concentration on HIC purification of romiplostim were analyzed by Box-Behnken design (Table 6). The significance of the proposed model for protein purity in this step was indicated by the F-value of 16.3 and a *p* value ≤ 0.05 (Supplementary Table 10). The proposed model for the protein recovery was expressed as a polynomial equation Eq. (5) in terms of three variables in which A, B, and C are coded values of pH, ammonium sulphate and, urea, respectively.

**Table 4:** Plackett-Burman matrix

**Run**	**pH**	**Time** ** (h)**	**Cystine** ** (mM)**	**Arginine** ** (mM)**	**EDTA ** **(mM)**	**Urea ** **(M)**	**Protein ** **(µg)**	**Refolding yield (%)**
1	6.5	40	5.0	100	1	0.5	660	1.2
2	6.5	40	0.5	500	1	2.0	660	80.0
3	8.5	16	5.0	100	5	2.0	660	67.2
4	8.5	40	0.5	500	5	0.5	165	85.35
5	6.5	16	0.5	100	1	0.5	165	43.2
6	8.5	40	5.0	100	1	2.0	165	84.0
7	8.5	16	5.0	500	1	0.5	165	80.0
8	8.5	40	0.5	100	5	0.5	660	84.1
9	6.5	16	5.0	500	5	0.5	660	4.45
10	6.5	16	0.5	100	5	2.0	165	69.8
11	8.5	16	0.5	500	1	2.0	660	75.5
12	6.5	40	5.0	100	5	2.0	165	12.9

Twelve runs were designed at various combinations of ‘high’ (+) and ‘low’ (-) values of refolding process parameters. Non-reduced 12% SDS-PAGE and densitometry analysis were used for calculating the refolding yield.

**Table 5:** The Box-Behnken design matrix with 15 runs and the corresponding output response (process impurity, product yield, and oxidized impurities)

**Run**	**pH**	**Cystine (mM)**	**Time (h)**	**Process impurity (%)**	**Refolding yield (%)**	**Oxidized impurity (%)**
1	9.5	2.5	48	22.8	59.2	18.0
2	7.5	1.5	24	51.0	30.7	18.3
3	9.5	0.5	48	33.4	50.3	16.3
4	8.5	2.5	24	16.5	65.0	15.0
5	8.5	2.5	72	4.6	85.8	9.6
6	7.5	0.5	48	33.5	42.7	23.8
7	7.5	1.5	72	14.7	78.8	6.5
8	7.5	2.5	48	17.2	74.1	8.7
9	8.5	0.5	24	44.0	36.0	20
10	9.5	1.5	24	37.1	46.0	16.9
11	8.5	1.5	48	36.4	46.5	17.1
12	8.5	1.5	48	27.5	54.7	17.8
13	9.5	1.5	72	6.9	80.6	12.5
14	8.5	1.5	48	27.9	52.2	19.9
15	8.5	0.5	72	41.3	43.6	15.1

Eq. (5):

Protein recovery=35.33-2.2A-2.61B+7.69C-1.6AB-0.85AC-0.425BC-11.98A^2^-10.7B^2^-16.3C^2^

**Fig. 4:**
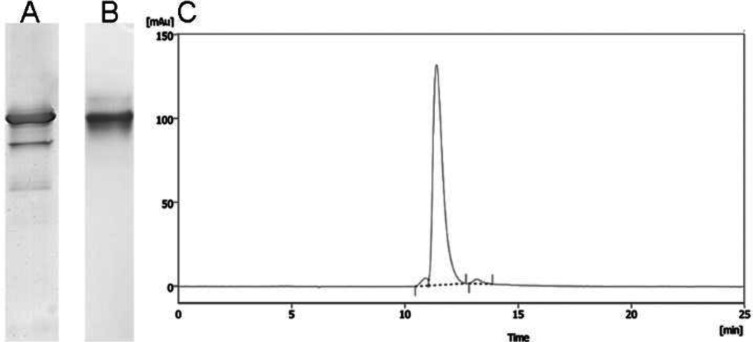
Purity analysis of romiplostim. SDS-PAGE analysis of the (A) refolded protein purified by HIC (experiment no. 4, Supplementary Fig. 4) and (B) subsequent (final) purification of romiplostim by preparative SEC; (C) analytical size-exclusion chromatography of the protein purified by preparative SEC Superdex 75

The coefficient of determination (R^2^: 0.96) identifies the goodness of fit for the model. The urea concentration was identified as a CPP and had the potential to influence protein recovery. HMW% was assessed in the selected experiments with the highest protein recovery and purity (Supplementary Fig. 8 and Supplementary Table 11). The optimized conditions (600 mM ammonium sulphate, 1.25 M urea, and pH 5.5) led to the minimum amount of HMW%. The HIC elution from the optimal condition was directly loaded on a Superdex 75 (Hiload^TM^ 16/600, Cytiva, Sweden) column with isocratic mode. The purity of the resultant protein was analyzed by analytical Size exclusion High performance chromatography (Fig. 4).

Potency assay

**Fig. 5:**
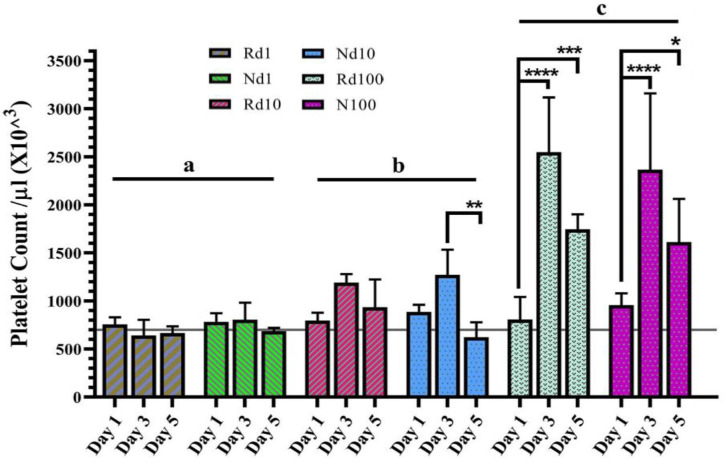
Comparison of dose-response effect of romiplostim and Nplate® in increasing platelet count in mice. Three sets of five female BALB/c mice (8-9 weeks old) in each group received a single subcutaneous dose of romiplostim or Nplate®. Blood samples were collected preorbital sinus from each set on days 1, 3, and 5. There was no significant difference in the platelet count romiplostim different dose compared to Nplate, indicated by the letter a, b and c. There was a significant intragroup difference in various doses of romiplostim and Nplate, shown by asterisks. Rd1, Rd10, and Rd100, romiplostim dose 1, 10, and 100 μg/kg; Nd1, Nd10, and Nd100, Nplate® dose 1, 10, and 100 μg/kg, respectively

The mean baseline blood platelet count of control mice was 700 × 10^3^/µl. No significant effect on platelet count occurred in mice receiving 1 μg/kg of romiplostim or Nplate^®^. Enhanced platelet count was observed in the groups receiving higher doses of both drugs (Fig. 5). In the mice receiving 10 and 100 μg/kg of both drugs, the platelet counts reached the highest level three days after subcutaneous administrations. The one-way ANOVA analysis revealed no statistically significant differences between the groups received romiplostim and Nplate^®^ (Supplementary Table 12). There was a significant intragroup difference in various doses of romiplostim and Nplate^®^, which is indicated by asterisks in Figure 5.

## DISCUSSION

**Fig. 6:**
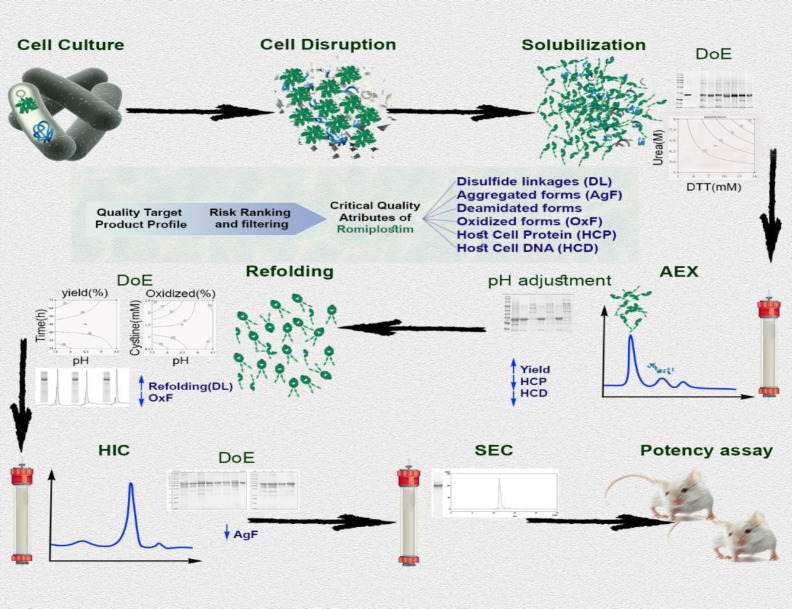
An overview of QbD approach used in romiplostim downstream processing development

While the QbD approach was successfully used to optimize the downstream process of recombinant proteins^[^^5^^,^^21^^]^, there is no predefined strategy for implementing QbD in pharmaceutical process development^[^^22^^,^^23^^]^. In the current case, a risk analysis was carried out to identify the CQAs of romiplostim. The risk study determined HMW aggregation, host cell proteins, host cell DNAs, oxidized forms, deamidation forms, and the right disulfide connections as CQAs. CQAs were measured in each step according to the logic of that step. It was impossible to measure the deamidation forms of romiplostim due to the citable method for deamidated romiplostim forms. To optimize the main downstream steps, the experimental design was performed by DoE, and the relevant process parameters were examined. Information from experimental designs was useful to identify CPPs, which had important effects on the relevant CQAs in each step (Fig. 6). 

In the solubilization of IBs, the concentration of urea, DTT, and the incubation time were selected to be optimized by Box-Behnken method. Urea as a chaotropic agent in the solubilization buffer is frequently reported for the denaturation of a wide range of proteins^[^^24^^,^^25^^]^. DTT is used as a reducing agent to break all the disulfide bonds that may be created during IBs formation or isolation. In fact, the optimization of solubilization step was reported in the manufacturing processes of different proteins. Mechin *et al.*^[^^26^^]^ examined the effect of urea and DTT concentrations. They reported that solubilization was more efficient at 5 or 7 M urea and 20 mM DTT, and an increase in DTT concentration did not produce any noticeable effect. Freydell *et al.*^[^^27^^]^ studied the solubilization behavior of a model protein using a statistically designed experiment. They found that in the alkaline pH, the higher urea concentration and DTT positively affects the amount of soluble protein. The highest solubilization yield of romiplostim would be achieved by 16 mM DTT and 8 M urea for 70 min, according to the provided model (Eq. 1). This maximum predicted yield was not much different from the maximum response of the actual experiment (Table 2, run 9) in which the same concentration of urea and DTT was used but within 52.5 minutes. 

**Table 6:** The Box-Behnken design matrix with 15 runs and the analyzed responses (protein recovery and protein purity)

**Run**	**pH**	**Ammonium sulfate (mM)**	**Urea (M)**	**Protein recovery (%)**	**Purity (%)**
1	8.5	1250	1.25	2.74	45.6
2	5.5	925	2.5	15.8	82.3
3	7	925	1.25	32.46	66.6
4	5.5	600	1.25	19.04	86
5	5.5	1250	1.25	13.01	83.3
6	7	600	2.5	17.24	78.9
7	8.5	925	2.5	12.41	64.8
8	7	925	1.25	34.64	71.2
9	8.5	600	1.25	15.53	72.1
10	7	925	1.25	38.86	80.5
11	7	1250	2.5	15.76	70
12	7	600	0	0	-
13	5.5	925	0	0	-
14	8.5	925	0	0	-
15	7	1250	0	0	-

Before refolding, a flowthrough mode AEX chromatography was used to reduce the host cell contaminants. Stone *et al.*^[^^28^^]^*.* reported the impacts of the process parameters, such as pH, conductivity, and the potential binding competition between HCP and HCD in a selected anion exchange media flowthrough. They showed that increasing the host cell proteins had no effect on the DNA clearance capability of the anion exchange media, probably because that some basic subpopulations of HCPs were not bound to the column. The main single parameter examined to achieve an effective removal of both HCP and HCD by Q Sepharose in flowthrough mode was pH (Table 3). A significant HCP and HCD clearance with acceptable protein recovery was observed at pH 6.8.

Since several factors influence protein refolding, a screening DoE was used to determine the CPPs. The cystine/cysteine ratio and pH were determined as CPPs, and incubation time (in a broader range) was selected to be optimized. The refolding yield was found to be significantly improved in alkaline pH. The thiol groups are reactive in alkaline conditions owing to their pKa values (pKa = 8.9)^[^^29^^]^. The increased disulfide bond formation tendency at alkaline pH explains the positive effect of pH on refolding yield. Wang *et al.*^[^^30^^]^ identified a “basic buffer” with the redox shuffling system (cystine/cysteine), which significantly increased the refolding rate of IL-17, while had little impact on its refolding yield. The effect of various ratios of cystine/cysteine on correct refolding of recombinant G-CSF was also evaluated by Tiwari *et al.*^[^^31^^]^. They found the optimized concentrations which were 1 mM for cystine and 2 mM for cysteine. A full factorial DoE for refolding optimization of a therapeutic fusion protein by dilution method was also reported. The refolding pH, concentrations of the solubilized IBs, urea, cystine, and DTT were identified as CPPs. The final model delivered a refolding yield of >77% and an oxidized impurity of <15%^[^^29^^]^. Our results showed an 85% refolding yield by pH 8, cystine/cysteine ratio of 0.5, and incubation time of 72 h. The common problem with Fc-fusion proteins is the formation of HMW aggregate and low molecular weight species^[^^32^^]^. HIC was used to remove product-related impurities, including misfolded protein of interest, aggregates, and fragments^[^^33^^]^. Jiang *et al.*^[^^7^^]^ reported that the amount of protein loading and resin hydrophobicity were CPPs in a HIC purification step of a fusion protein. They achieved an HMW aggregate of ≤2.5% and a yield of ≥40% by optimizing the stated process parameters. The concentration of urea, ammonium sulphate, and pH selected as process parameters in HIC were optimized by Box-Behnken method. Urea concentration was determined as CPP. Based on the purity and protein recovery, HMW level of ≤2% and a recovery yield ≥38% were achieved. A broad range of parameters, such as urea is recommended to improve the recovery rate. SEC was used for final polishing (Fig. 4), and the biological activity of the purified sample was measured *in vivo*. The platelet counts peaked three days after subcutaneous administrations in the mice receiving 10 and 100 μg/kg of romiplostim and Nplate^®^. As expected, platelet increase was transient and returned to the normal range after several days.

In general, a successful strategy to optimize the different purification steps of romiplostim was explained. The CQAs of romiplostim was defined and examined selectively according to the targeted outputs of each step and the CQA measurement practicability on the intermediate product, as well. The design space was sketched using DoE to define CPPs and determine their relations to the optimized conditions. Hence, a reliable operating strategy was identified for each downstream process step, enabling a higher yield while ensuring acceptable product quality.

### Ethical statement

The mouse experiments in this study were approved by the Ethics Committee of Pasteur Institute of Iran, Tehran (ethical code: IR.PII.REC.1399.003). All applicable international, national, and/or institutional guideline(s) for the care and use of animals were followed.

### Data availability

All the data supporting the findings of this study are available within the article and its supplementary materials.

### Author contributions

SP: conceived and designed experiments, performed risk assessment and data analysis, performed purification experiment and related analyses, performed biological activity analysis, and wrote the manuscript; FT: conceived and designed experiments, performed risk assessment and data analysis, designed the experimental strategy, and supervised analysis; HA: performed purification experiment and related analyses; MA: performed purification experiment and related analyses; PFE: designed the experimental strategy and supervised analysis; MG: conceived and designed experiments, performed biological activity analysis, designed the experimental strategy, and supervised analysis; BV: wrote the manuscript, designed the experimental strategy, and supervised analysis. 

### Conflicts of interest

The authors declare that they have no known competing financial interests or personal relationships that could have appeared to influence the work reported in this paper.

### Funding/support

This work was supported by Pasteur Institute of Iran (grant no. BP-9365) and Iran National Science Foundation (INSF; grant no. 90007372).

## Supplementary Materials


