# Supplementary material for: Quality by Design in Downstream Process Development of Romiplostim
Source: Iran Biomed J. 2022 Oct 30;26(6):414–25. doi: 10.52547/ibj.3790 (PMC9841220; doi:10.52547/ibj.3790)
Supplement: Supplementary file 1 [file ibj-26-414-s1.pdf]

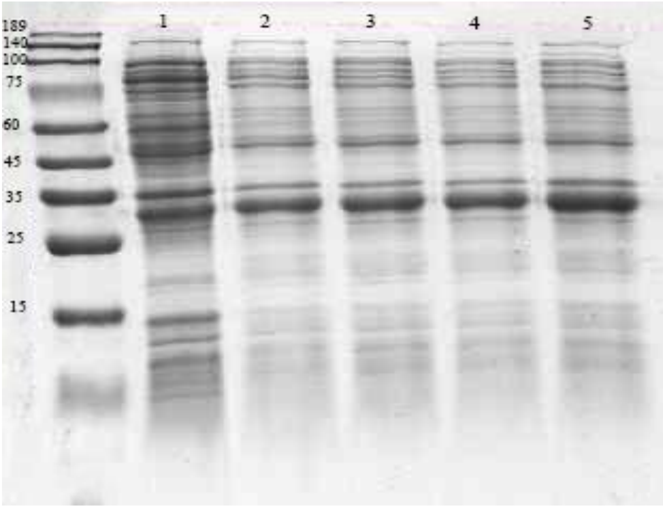

Supplementary Fig. 1. Reduced 12% SDS-PAGE of romiplostim expression. The gel was stained with Coomassie brilliant blue (G-250). Expression after 1 h (lane 1), 3 h (lane 2), 4 h (lane 3), 4 h (lane 4), and 6 h (lane 5).

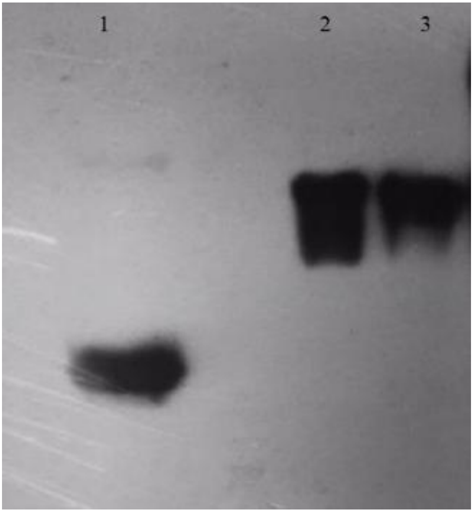

Supplementary Fig. 2. Western blot analysis of romiplostim. Lane 1, reduced form of romiplostim with a molecular weight of about 30 kDa; lane 2, non-reduced form of romiplostim with a molecular weight of about 60 kDa; lane 3, non-reduced Nplate as reference standard with a molecular weight of about 60 kDa.

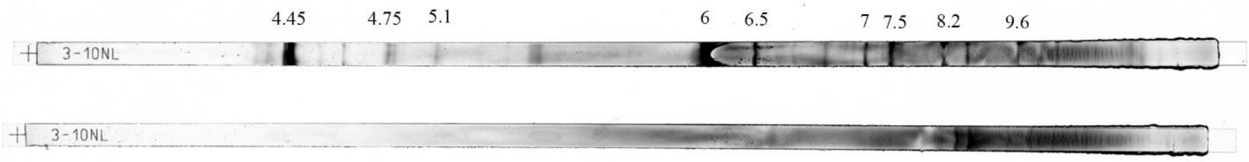

Supplementary Fig. 3. Seventeen-centimeter isoelectric focusing (IEF) strips (IEF strips pH 3–10 linear; Amersham Biosciences, UK) reswelled with 350 mL of urea lysis buffer, freshly spiked with 2% pharmalytes (pH 3–10; Amersham Biosciences) and 1% dithiothreitol, at room temperature overnight. Nplate (15 µL) as reference product loaded on the IEF strips was electrophoresed.

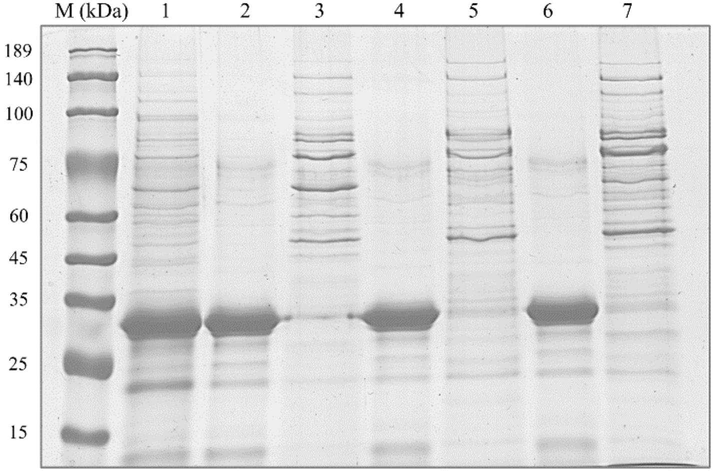

Supplementary Fig. 4. Non-reduced 12% SDS-PAGE of anion exchange chromatography chromatography. The gel was stained with Coomassie brilliant blue (G-250). Lane 1, solubilization sample; lane 2, FT sample (pH 6.8); lane 3, elution sample (pH 6.8); lane 4, FT sample (pH 7.4); lane 5, elution (pH 7.4); lane 6, FT sample (pH 8); lane 7, elution (pH 8). FT, flow-through

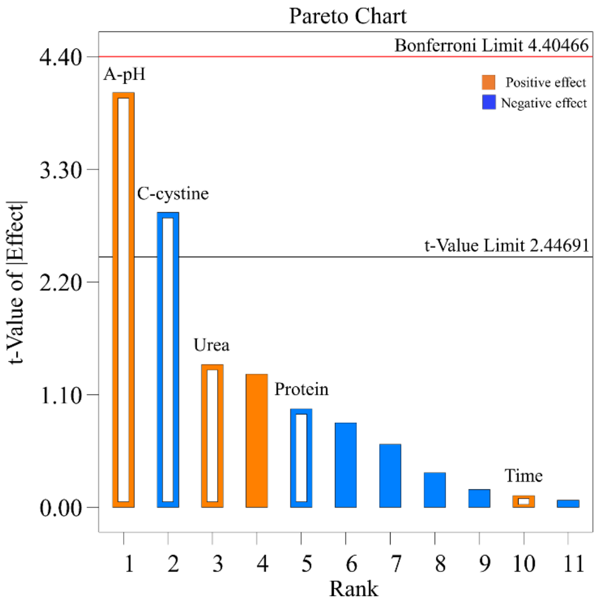

Supplementary Fig. 5. The positive and negative process parameters (PPs) on the refolding yield. The *p* of the pH and cystine was 0.0067 and 0.028, respectively. pH and cystine were known as CPPs. Incubation time, urea concentration, and protein concentration did not have any significant effect on the refolding yield at the studied range.

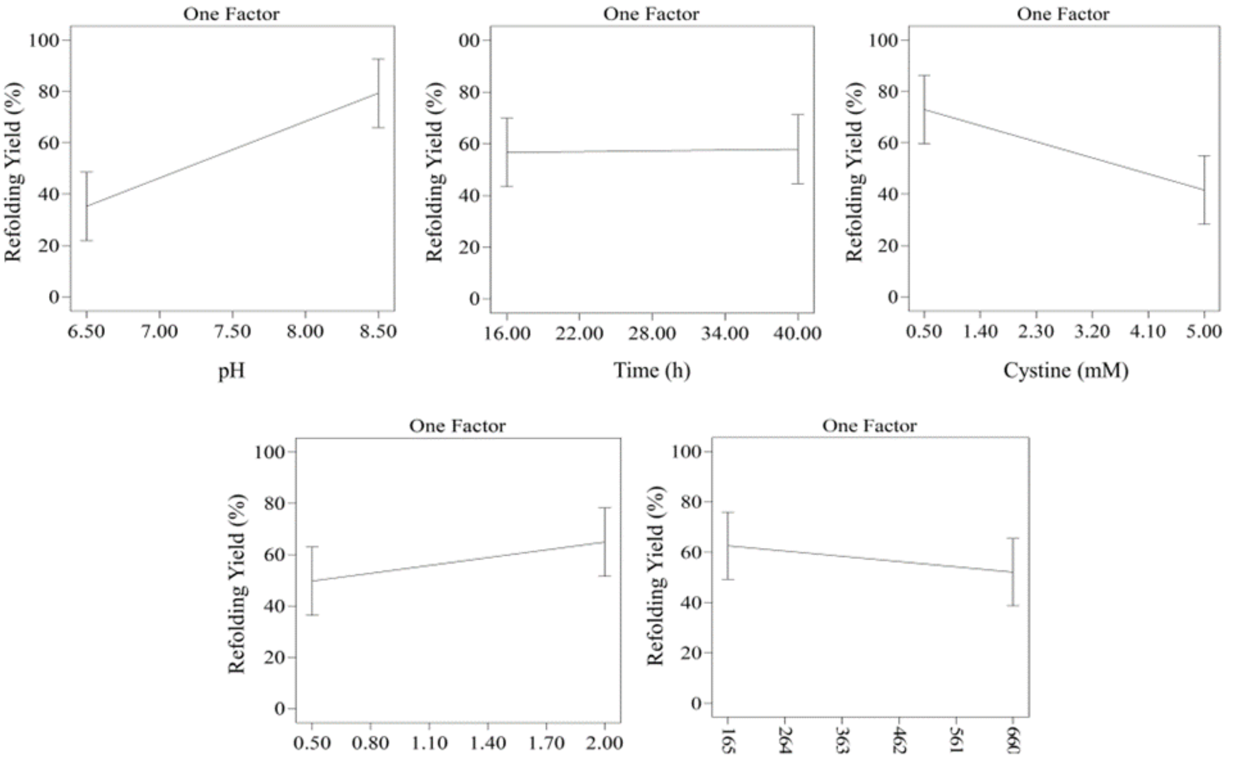

Supplementary Fig. 6. The process parameters with positive or negative effects on the refolding yield. pH and cysteine concentration were considered critical process parameters. The *p* of the effect of pH and cystine concentrations on the refolding yield was 0.0067 and 0.02, respectively. Incubation time, urea concentration, and protein concentration did not significantly affect the refolding yield at the studied range.

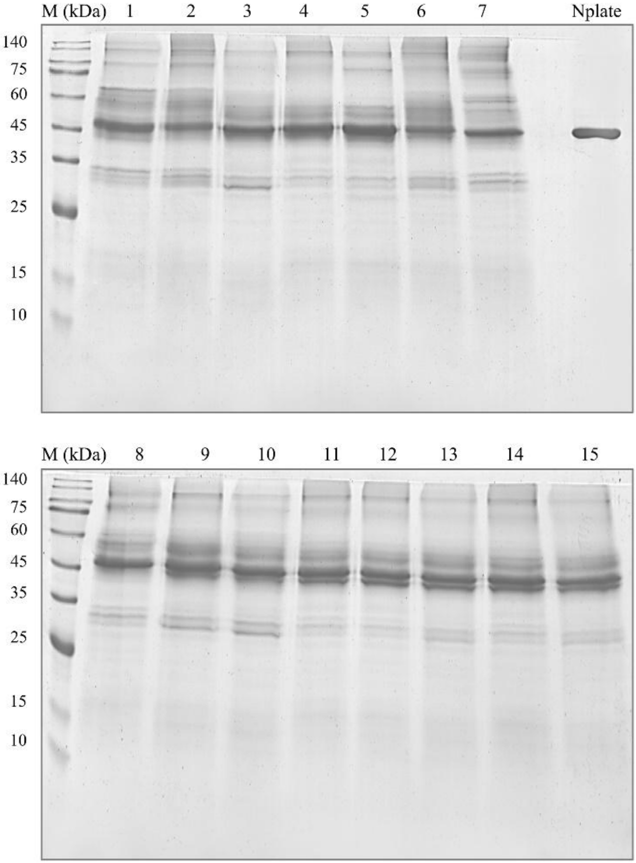

Supplementary Fig. 7. SDS-PAGE analysis of the refolding from 15 designed experiments by Box-Behnken. The gels were stained with Coomassie brilliant blue (G-250).

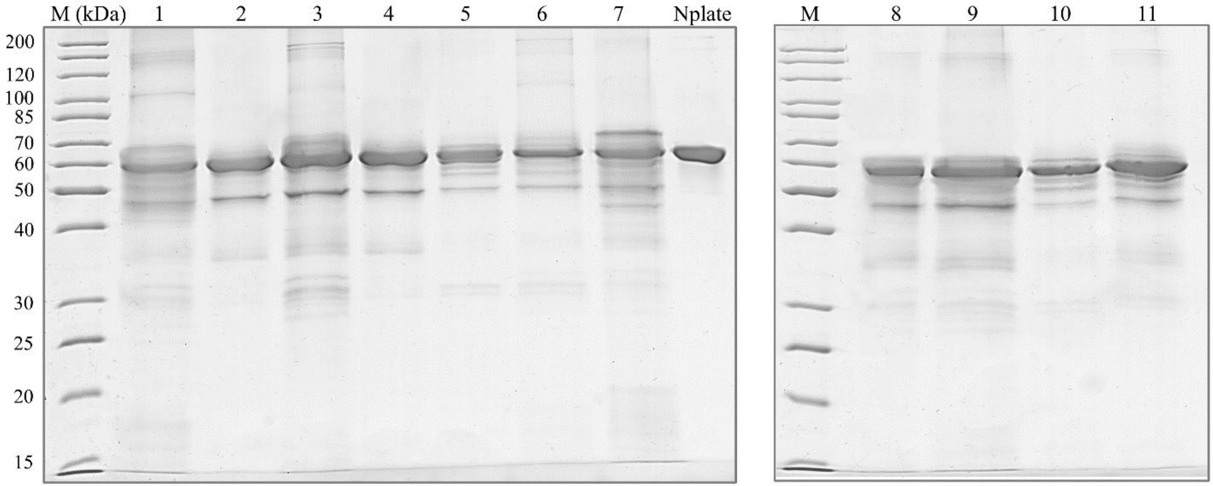

Supplementary Fig. 8. SDS-PAGE analysis of hydrophobic interaction chromatography from 15 designed experiments (Box-Behnken). The protein recovery under 10% was not loaded on the gel. The gel was stained with Coomassie brilliant blue (G-250).

Supplementary Table 1. Impact definition and scale

(Score)	Biological activity/efficacy	PK/PD	Immunogenicity	Safety
Very high (20)	Very significant change	Significant change on PK	ATA detected and confers limits on safety	Irreversible AEs
High (16)	Significant change	Moderate change with impact on PD	ATA detected and confers limits on efficacy	Reversible AEs
Moderate (12)	Moderate change	Moderate change with no impact on PD	ATA detected with *in vivo* effect that can be managed	Manageable AEs
Low (4)	Acceptable change	Acceptable change with no impact on PD	ATA detected with minimal *in vivo* effect	Minor, transient AEs
None (2)	No change	No impact on PK or PD	ATA not detected or ATA detected with no relevant in vivo effect	No AEs

PK, pharmacokinetics; PD, pharmacodynamics; AE, adverse event; ATA, anti-therapeutic antibody

Supplementary Table 2. Uncertainty definition and scale

**Uncertainty (score)**	**Description (Variants and host-related impurities)**
7 (Very high)	No information (new variant)
5 (High)	Published external literature for variant in related molecule
3 (Moderate)	Nonclinical or *in vitro* data with this molecule. Data (nonclinical, *in vitro* or clinical) from a similar class of molecule
2 (Low)	Variant has been present in material used in clinical trials
1 (Very low)	Impact of specific variant established in clinical studies with this molecule

Supplementary Table 3. Design of experiment inputs: process parameters (PPs) and ranges for characterization studies of optimization solubilization

**PPs**	**Range**		
	**Low (-)**	**Middle (0)**	**High (+)**
Dithiothreitol (mM)	1	8.5	16
Urea (M)	5	6.5	8
Time (min)	15	52.5	90

Supplementary Table 4. Design of experiment inputs: process parameters and ranges evaluated during characterization studies of the refolding.

PPs	Range	
	**Low (-)**	**High (+)**
pH	6.5	8.5
Arginine (mM)	100.0	500.0
Cystine (mM)[a]	0.5	5.0
EDTA (mM)	1.0	5.0
Urea (M)	0.5	2.0
Protein (µg/ml)	165.0	660.0
Time (h)	16.0	40.0

[a]Cystine/cysteine ratio, in all experiments the concentration of cysteine was (5 mM).

Supplementary Table 5. Design of experiment inputs: process parameters (PPs) and ranges for characterization studies of optimization HIC.

**PPs**	**Range**		
	**Low (-)**	**Middle (0)**	**High (+)**
pH	5.5	7.0	8.5
Ammonium sulfate (mM)	600.0	925.0	1250.0
Urea (M)	0	1.25	2.5

Supplementary Table 6. The ANOVA results of the obtained model for solubilization step

**Source**	**Sum of Squares**	**df**	**Mean square**	**F-value**	***p* value**	
Model	1.80	9	0.2005	24.91	0.0012	significant
A-DTT	0.0578	1	0.0578	7.18	0.0438	
B-urea	1.54	1	1.54	191.47	<0.0001	
C-time	0.0594	1	0.0594	7.39	0.0419	
AB	0.0114	1	0.0114	1.42	0.2866	
AC	0.0001	1	0.0001	0.0177	0.8992	
BC	0.0029	1	0.0029	0.3637	0.5728	
A^2	0.0138	1	0.0138	1.71	0.2475	
B^2	0.0730	1	0.0730	9.07	0.0297	
C^2	0.0620	1	0.0620	7.71	0.0391	
Residual	0.0402	5	0.0080			
Lack of fit	0.0272	3	0.0091	1.40	0.4429	not significant
Pure error	0.0130	2	0.0065			
Cor total	1.84	14				

DTT, dithiothreitol

Supplementary Table 7. The ANOVA results of the obtained Plackett-Burman model for refolding

Source	Sum of squares	df	Mean square	F-value	*p* value	
Model	9810.5	5	1962.17	5.52	0.0302	Significant
A-pH	5834.3	1	5834.43	16.41	0.0067	
B-time	4.56	1	4.56	0.018	0.9135	
C-cystine	2951.0	1	2951.60	8.30	0.0280	
F-urea	691.60	1	691.60	1.94	0.2126	
G-protein	328.65	1	328.65	0.922	0.3735	
Residual	2133.6	6	355.61			
Cor total	11944.5	11				

Supplementary Table 8. The ANOVA results of the obtained box-Behnken model for refolding step

**Source**	**Sum of squares**	**df**	**Mean square**	**F-value**	***p* value**	
Model	3148.83	9	349.87	6.60	0.0256	significant
A-pH	27.38	1	27.38	0.5167	0.5044	
B-cystine	1554.03	1	1554.03	29.33	0.0029	
C-time	935.28	1	935.28	17.65	0.0085	
AB	126.56	1	126.56	2.39	0.1829	
AC	362.90	1	362.90	6.85	0.0473	
BC	43.56	1	43.56	0.8221	0.4062	
A^2	0.4741	1	0.4741	0.0089	0.9283	
B^2	95.41	1	95.41	1.80	0.2374	
C^2	7.07	1	7.07	0.1333	0.7299	
Residual	264.94	5	52.99			
Lack of fit	229.62	3	76.54	4.33	0.1932	not significant
Pure error	35.33	2	17.66			
Cor total	3413.77	14				

The output was refolding yield.

Supplentary Table 9. The ANOVA results of the obtained box-Behnken model for refolding step

Source	Sum of squares	df	Mean square	F-value	*P* value	
Model	293.27	9	32.59	15.33	0.0039	significant
A-pH	5.12	1	5.12	2.41	0.1814	
B-cystine	71.40	1	71.40	33.59	0.0022	
C-time	87.78	1	87.78	41.29	0.0014	
AB	70.56	1	70.56	33.19	0.0022	
AC	13.69	1	13.69	6.44	0.0520	
BC	0.0625	1	0.0625	0.0294	0.8706	
A^2	7.99	1	7.99	3.76	0.1103	
B^2	0.0339	1	0.0339	0.0160	0.9044	
C^2	38.90	1	38.90	18.30	0.0079	
Residual	10.63	5	2.13			
Lack of fit	6.38	3	2.13	1.00	0.5347	not significant
Pure error	4.25	2	2.12			
Cor total	303.90	14				

The output was oxidized form.

Supplementary Table 10. The ANOVA results of the obtained box-Behnken model for HIC step

Source	Sum of squares	df	Mean square	F-value	*p* value	
Model	2270.24	9	252.25	16.30	0.0034	significant
A-pH	38.72	1	38.72	2.50	0.1746	
B-ammonium sulfate	54.60	1	54.60	3.53	0.1192	
C-urea	472.78	1	472.78	30.54	0.0027	
AB	10.24	1	10.24	0.6615	0.4530	
AC	2.89	1	2.89	0.1867	0.6837	
BC	0.7225	1	0.7225	0.0467	0.8375	
A^2	529.85	1	529.85	34.23	0.0021	
B^2	423.06	1	423.06	27.33	0.0034	
C^2	981.51	1	981.51	63.41	0.0005	
Residual	77.40	5	15.48			
Lack of fit	56.11	3	18.70	1.76	0.3827	not significant
Pure error	21.29	2	10.64			
Cor total	2347.64	14				

The output was protein recovery

Supplementary Table 11. Comparison high molecular weight (HMW) aggregate of four selected design experiments of hydrophobic interaction chromatography optimization

Run	pH	Ammonium sulfate (mM)	Urea (M)	HMW %
2	5.5	600	1.25	0.1
4	5.5	925	2.50	0.6
6	7.0	600	2.50	2.0
10	7.0	925	1.25	0.6

Supplementary Table 12. Female BALB/c mice (8-9 weeks of age; n = 5/time point) receiving a single subcutaneous dose of romiplostim and Nplate 1, 10, and 100 μg/kg in mice

**Nplate**			**Romiplostim**			**ANOVA**
**Dose value (μg/kg)**	**Day**	**Mean response (10^3/μl)**	**Dose value (μg/kg)**	**Day**	**Mean response (10^3/μl)**	$p \leq 0.05$
1	1	783.33	1	1	756.66	0.998
10	1	886.66	10	1	796.66	0.697
100	1	776.66	100	1	636.66	0.280
1	3	723.00	1	3	682.50	0.999
10	3	1289.33	10	3	1190.00	0.998
100	3	2793.33	100	3	2546.66	0.449
1	5	686.66	1	5	666.66	1.000
10	5	733.33	10	5	739.66	1.000
100	5	1756.00	100	5	1747.50	1.000

The platelet-increasing effect was evaluated based on the platelet count at baseline up to 10 days after administration (1, 3, and 5 days after administration).
